# A Personalized Multi-Channel FES Controller Based on Muscle Synergies to Support Gait Rehabilitation after Stroke

**DOI:** 10.3389/fnins.2016.00425

**Published:** 2016-09-16

**Authors:** Simona Ferrante, Noelia Chia Bejarano, Emilia Ambrosini, Antonio Nardone, Anna M. Turcato, Marco Monticone, Giancarlo Ferrigno, Alessandra Pedrocchi

**Affiliations:** ^1^Neuroengineering and Medical Robotics Laboratory, Department of Electronics, Information and Bioengineering, Politecnico di MilanoMilan, Italy; ^2^Physical Medicine and Rehabilitation Unit, Scientific Institute of Lissone, Fondazione Salvatore Maugeri (IRCCS)Lissone, Monza Brianza, Italy; ^3^Posture and Movement Laboratory, Division of Physical Medicine and Rehabilitation, Scientific Institute of Veruno, Fondazione Salvatore Maugeri (IRCCS)Veruno, Novara, Italy; ^4^Department of Translational Medicine, University of Eastern PiedmontNovara, Italy; ^5^Department of Public Health, Clinical and Molecular Medicine, University of CagliariCagliari, Italy

**Keywords:** functional electrical stimulation, stroke rehabilitation, locomotion, treadmill, muscle synergies

## Abstract

It has been largely suggested in neuroscience literature that to generate a vast variety of movements, the Central Nervous System (CNS) recruits a reduced set of coordinated patterns of muscle activities, defined as muscle synergies. Recent neurophysiological studies have recommended the analysis of muscle synergies to finely assess the patient's impairment, to design personalized interventions based on the specific nature of the impairment, and to evaluate the treatment outcomes. In this scope, the aim of this study was to design a personalized multi-channel functional electrical stimulation (FES) controller for gait training, integrating three novel aspects: (1) the FES strategy was based on healthy muscle synergies in order to mimic the neural solutions adopted by the CNS to generate locomotion; (2) the FES strategy was personalized according to an initial locomotion assessment of the patient and was designed to specifically activate the impaired biomechanical functions; (3) the FES strategy was mapped accurately on the altered gait kinematics providing a maximal synchronization between patient's volitional gait and stimulation patterns. The novel intervention was tested on two chronic stroke patients. They underwent a 4-week intervention consisting of 30-min sessions of FES-supported treadmill walking three times per week. The two patients were characterized by a mild gait disability (walking speed > 0.8 m/s) at baseline. However, before treatment both patients presented only three independent muscle synergies during locomotion, resembling two different gait abnormalities. After treatment, the number of extracted synergies became four and they increased their resemblance with the physiological muscle synergies, which indicated a general improvement in muscle coordination. The originally merged synergies seemed to regain their distinct role in locomotion control. The treatment benefits were more evident for one patient, who achieved a clinically important change in dynamic balance (Mini-Best Test increased from 17 to 22) coupled with a very positive perceived treatment effect (GRC = 4). The treatment had started the neuro-motor relearning process also on the second subject, but twelve sessions were not enough to achieve clinically relevant improvements. This attempt to apply the novel theories of neuroscience research in stroke rehabilitation has provided promising results, and deserves to be further investigated in a larger clinical study.

## Introduction

The rehabilitation of neurological patients strongly benefits of task-oriented, immersive, repetitive exercises when the patient experiences an enriched, augmented sensorial feedback. Indeed, such interventions stimulate the activity-dependent plasticity of the Central Nervous System (CNS) thus facilitating motor relearning (Ting et al., 2015). Activity-dependent plasticity is further enhanced when Functional Electrical Stimulation (FES) is synchronized with task-oriented volitional exercises (Sheffler and Chae, 2007; Chae et al., 2008; Gandolla et al., 2014, 2016; Kafri and Laufer, 2015), such as cycling (Ferrante et al., 2008; Ambrosini et al., 2011, 2012) and walking (Kesar et al., 2009; Embrey et al., 2010; Sabut et al., 2010, 2011; Daly et al., 2011; Kim et al., 2012). Indeed, the increased afferent feedback provided by FES modulates motor cortex function and excitability to facilitate recovery (Ridding et al., 2000; Gandolla et al., 2014, 2016).

The first FES-based gait systems were designed for the treatment of foot drop, combining single-channel stimulation of the peroneal nerve with a pressure sensor to detect the initial contact of the foot with the ground (Melo et al., 2015). Since then, multi-channel FES strategies have been proposed and tested in stroke patients (Kesar et al., 2009; Ambrosini et al., 2010; Embrey et al., 2010; Sabut et al., 2010, 2011; Daly et al., 2011; Kim et al., 2012). However, in all FES-based gait systems, only the two main gait phases (i.e., the stance and swing phase) were detected and used to trigger the stimulation of the different muscles involved in the movement. The stimulation waveforms were mainly trapezoidal (Melo et al., 2015). These waveforms use a ramp up of stimulation amplitude at a constant pulse width to avoid sudden and jerky muscle responses both in the agonist and antagonist muscles, and a ramp down to avoid a sudden and unpleasant slap of the foot on the ground. Biomimetic stimulation controllers, which modulate the stimulation amplitude based on physiological EMG activations, were proposed for a single-channel drop-foot stimulator and were tested on a single patient, resulting to be more efficient than trapezoidal profiles (O'Keeffe et al., 2003). Biomimetic multi-channel FES systems have shown promising therapeutic benefits when applied in stroke patients during cycling (Ferrante et al., 2008; Ambrosini et al., 2011, 2012). However, to the authors' knowledge, a biomimetic multi-channel FES system has not yet been proposed and tested during gait.

To design a biomimetic FES controller, it is essential to mimic the neural solutions adopted by the CNS to generate movements. It has been largely suggested in neuroscience literature that in order to generate a vast variety of movements, the CNS recruits a reduced set of coordinated patterns of muscle activities, defined as muscle synergies or motor modules (d'Avella et al., 2003; d'Avella and Bizzi, 2005). Further, a study on spinalized rats has provided experimental evidence that the CNS simplifies the complexity and high dimensionality of neural commands and mechanical outputs by means of a modular organization at the neuromuscular level (Mussa-Ivaldi and Bizzi, 2000).

The concept of muscle synergy has been formalized with a mathematical model based on factorization algorithms that decompose the EMG signals into the product of two components. The weighting component reveals the muscle composition of each synergy and the relative level of activation of each muscle, whereas the temporal component reflects the activation timing of each synergy throughout the execution of the movement. Each muscle synergy contributes to the mechanical output needed to generate task-specific biomechanical functions, also called biomechanical correlates (Lacquaniti et al., 2012). Many studies on physiological gait have agreed in the definition of four synergies as responsible of the main biomechanical correlates on healthy subjects (Clark et al., 2010; Barroso et al., 2014; Routson et al., 2014):

Synergy 1 (weight acceptance): activation of the hip and knee extensors during early stance that is associated with weight acceptance;

Synergy 2 (push off): activation of the ankle plantar-flexors in late stance that is associated with forward propulsion;

Synergy 3 (foot clearance): activation of the rectus femoris and the tibialis anterior during early stance and early swing, which provides foot dorsi-flexion immediately after heel strike and ground clearance of the foot, respectively;

Synergy 4 (leg deceleration): activation of the hamstrings during late swing and early stance to decelerate the leg and propel the body.

An additional synergy can be found when the trunk muscles are also recorded (Ivanenko et al., 2005). Simulation studies have confirmed the validity of the biomechanical correlates of the muscle synergies (Neptune et al., 2009; Allen and Neptune, 2012).

Muscle synergies have been shown to be “fixed” because they are consistent across different subjects despite variability and noise in the neuro-musculo-skeletal system, but also “flexible” so that they can adapt to slight changes in the environment or be affected by pathologies and then modulated with rehabilitation training (Santello and Lang, 2014). For instance, during locomotion post-stroke individuals exhibit a reduced number of synergies in their paretic side due to the merging of motor modules that imply a non-functional muscle co-contraction reflecting walking dysfunctions (Bowden et al., 2010; Clark et al., 2010; Ting et al., 2015). It is likely that this reduction is caused by a lack of independence of the corticospinal drive to the spinal cord, which ultimately causes a poor muscle control.

Muscle-synergy analysis is currently considered a useful methodology to assess sensorimotor individual deficits (Safavynia et al., 2011). Further, it could be a potential ground upon which novel therapies aimed at enhancing motor relearning could be designed (Ting et al., 2015). In this scope, a FES training based on healthy muscle synergies has been recently proposed for a balance control task. However, the experimental apparatus was rather complex, discouraging its translation to the clinical practice (Galeano et al., 2014).

Our study merges the potentialities of FES-based gait treatments with the strength of muscle-synergy training approach. Indeed, this study was aimed at designing a personalized, biomimetic, multi-channel stimulation controller to support gait rehabilitation after stroke, integrating the following novel aspects:

1. the FES strategy is based on the physiological muscle synergies obtained during overground locomotion.
2. the FES strategy is personalized according to an initial locomotion assessment of the patient, and is used to properly activate impaired biomechanical correlates.
3. the FES strategy is mapped accurately on the altered gait kinematics taking advantage of a segmentation algorithm able to discriminate in real time between 6 gait phases (Chia Bejarano et al., 2015b), allowing a maximal synchronization between the subject's volitional gait and the stimulation patterns.

Preliminary results obtained from two chronic stroke patients with the proposed FES gait controller will be presented in order to show the potentiality of this novel intervention.

## Materials and methods

### The stimulation controller architecture

**Figure 1:**
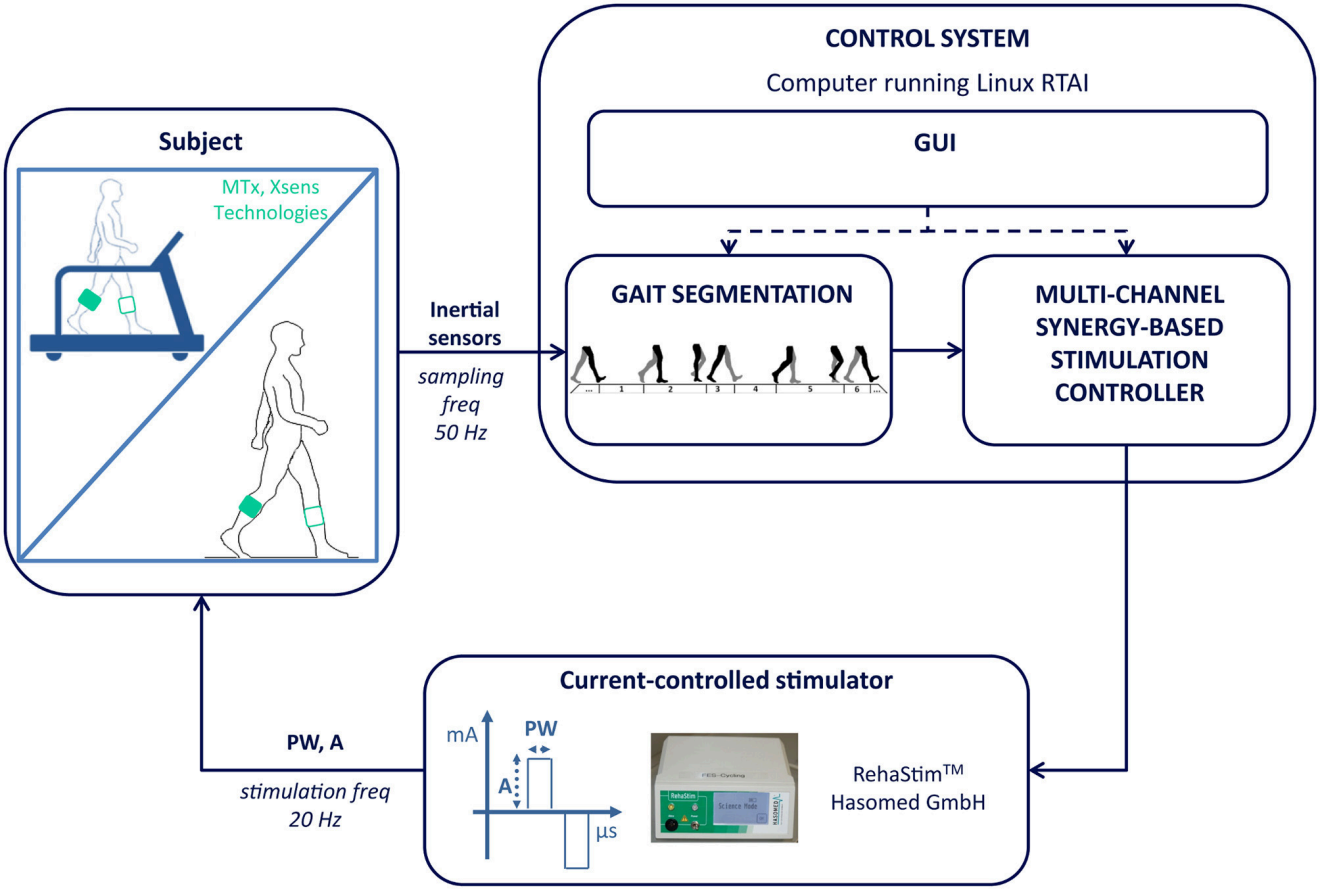
**The stimulation controller architecture**. In the control system block, real-time signals and non-real-time signals are indicated with solid and dashed arrows respectively. GUI, graphical user interface; freq, frequency; PW, pulse width; A, amplitude.

The FES-controller architecture includes the subject that can walk overground or on a treadmill, a PC running Linux RTAI, which hosts the whole control system, and a current-controlled stimulator (Rehastim®, Hasomed GmbH) delivering biphasic pulses to surface electrodes (Pals®, Axelgaard Manufacturing Co., Ltd.) placed on up to 8 muscles of the paretic leg. The subjects wear two inertial sensors (Mtx®, Xsens Technology), on both shanks, which provide a real-time kinematic measure used to accurately synchronize the stimulation to the gait cycle. The control system comprises a graphical user interface (GUI) implemented in Qt™software and two real-time applications. The GUI allows the therapist to customize the treatment on the single patient, to start, pause, and stop the treatment, to save data, and to access the stored data. The first real-time application of the control system is the *gait segmentation block* (see Figure 1), which receives the signals from the inertial sensors and estimates the Initial Contact (IC), the End Contact (EC), and the Mid-Swing (MS) gait events for each leg. This algorithm was adapted from Chia Bejarano et al. (2015b), in order to be used robustly also in a magnetically disturbed environment, and was validated on healthy subjects using the force-sensitive resistors as a gold standard (Chia Bejarano et al., 2015a). The algorithm demonstrated an excellent accuracy in detecting the IC and EC events (F1-score of 0.98 for the IC and 0.96 for the EC), and allows the detection of the following 6 gait phases: paretic double support, non-paretic initial swing, non-paretic terminal swing, non-paretic double support, paretic initial swing, paretic terminal swing (Figure 1).

**Figure 2:**
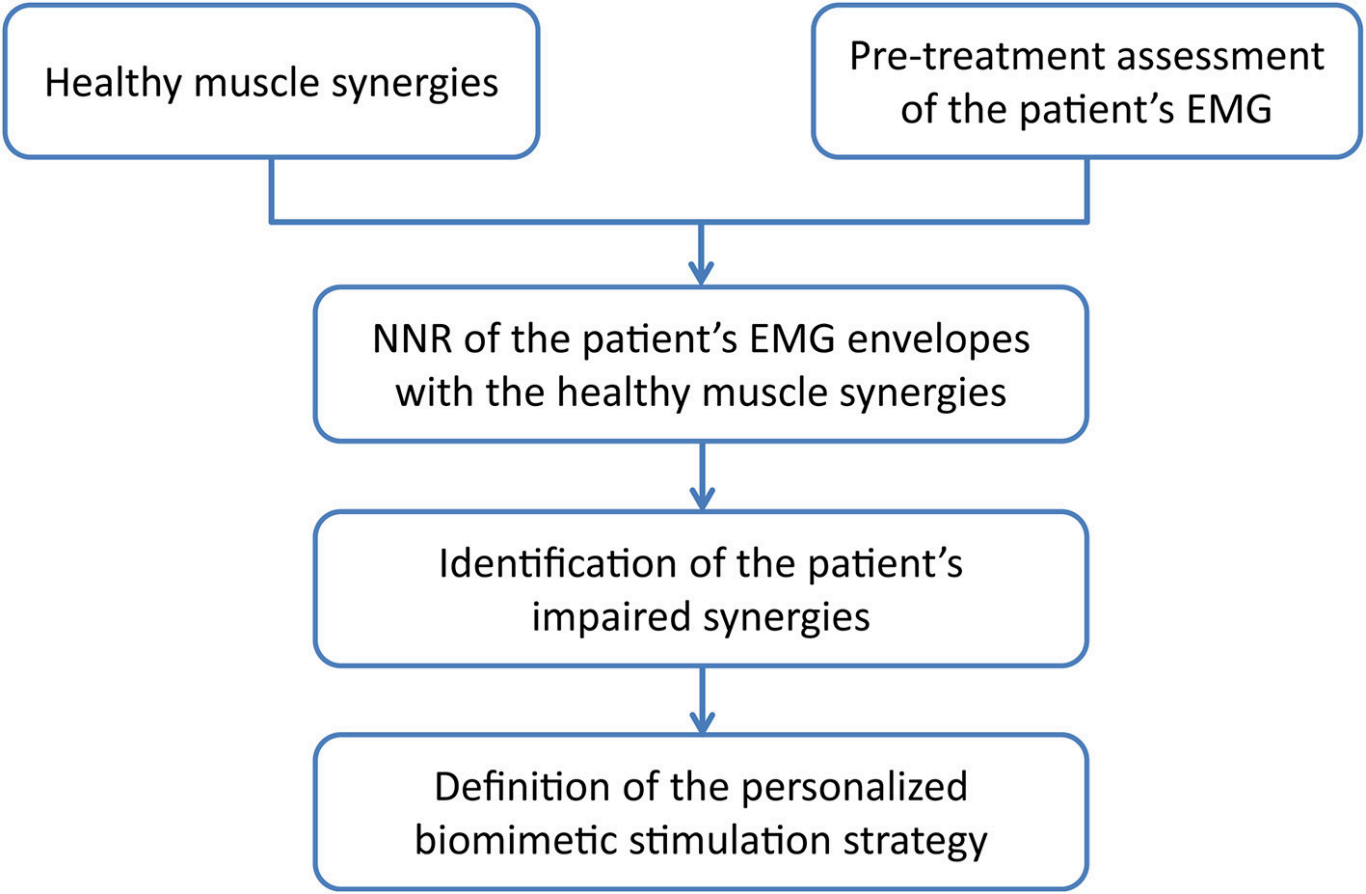
**The methodology used to define the personalized biomimetic stimulation strategy**. NNR, Non-Negative Matrix Reconstruction.

The second real-time application is the *multi-channel synergy-based stimulation controller* that is personalized on each patient following the steps reported in Figure 2 and detailed below.

#### Healthy muscle synergies

The starting point of the stimulation controller is the definition of a set of representative healthy muscle synergies. Thirteen healthy subjects (7 men and 6 women; age: 24.8 ± 1.3 years; height: 1.73 ± 0.11 m, weight: 60.8 ± 11.4 kg) volunteered to participate in this study. They were asked to walk overground and on a treadmill at their self-selected speed. The EMG was measured on the following muscles of the dominant leg: gluteus maximus (GM), rectus femoris (RF), vastus medialis (VM), hamstring medialis (HM), hamstring lateralis (HL), gastrocnemius medialis (MG), and tibialis anterior (TA). Kinematics data were acquired at 50 Hz by means of 2 inertial and magnetic sensors (MTx, Xsens) worn on both shanks (Chia Bejarano et al., 2015b).

The EMG signals were acquired at 1024 Hz, band-pass filtered (3rd-order Butterworth filter, cut-off frequencies of 40 and 400 Hz), rectified, and low-pass filtered (3rd-order Butterworth filter, cut-off frequency of 5 Hz) to obtain the EMG envelopes. Afterwards, the envelopes were segmented into single strides using the first contacts of the ipsilateral foot with the pavement (IC events). Then, each stride was normalized in time by interpolating the signals into 100 points, and in amplitude by dividing the EMG signals of each muscle by the median maximum value obtained across strides for each walking condition (treadmill and overground). After removing the initial acceleration and the final deceleration phases from each acquisition, 20 representative strides for each subject and condition were extracted as suggested by Oliveira et al. (2014). The non-negative matrix factorization (NMF) algorithm was applied separately to the 20 envelopes obtained for each subject and walking condition, in order to extract their muscle synergies (Lee and Seung, 1999). The quality of the factorization was measured by computing the variability accounted for (VAF) and the number of muscle synergies was chosen as the smallest number that allowed the reconstruction with a total VAF higher than 90%, or that did not improve the single-muscle VAF more than 5% when adding a new synergy (Clark et al., 2010). The individual muscle synergies of each walking condition were extracted using the most representative number of synergies obtained according to the chosen VAF criterion. Then, the weights of each individual muscle synergy were normalized to have a unitary norm, applying the corresponding transformation to their respective activations profiles, to maintain constant their product. For each walking condition, the average set of muscle synergies across subjects was calculated. To compare the healthy muscle synergies obtained during overground and treadmill walking, the mean and standard deviation of the following metrics were computed: (1) the similarity, i.e., the normalized scalar product between the weights (W) extracted in the two walking conditions; (2) the circular cross correlation between the activation profiles (H) extracted in the two conditions; (3) the lag in percentage of gait cycle in which the maximal circular cross correlation was obtained. If the two walking conditions were comparable in terms of muscle synergies, the healthy synergies extracted from the overground walking condition could be used both as a reference to evaluate the overground waking coordination of patients before and after treatment, and to build the biomimetic stimulation strategy to be applied during treadmill training.

#### Pre-treatment assessment of the patient's EMG

Before starting the treatment, the patient was asked to walk overground and the surface EMG activation signals were measured on eight muscles of the paretic leg following the same protocol described in the previous paragraph for healthy subjects. Analogously, the signal processing procedure described above was used to obtain the EMG envelopes.

#### NNR of the patient's EMG envelopes with the healthy muscle synergies

The mean set of weights (W_HEALTHY_) and activation profiles (H_HEALTHY_) obtained during overground walking in healthy subjects were used to perform the two Non-Negative Matrix Reconstructions (NNR) of the EMG envelopes obtained on the paretic side during the patient's pre-treatment assessment. The NNR was applied by fixing the synergy vectors as W_HEALTHY_ and letting only the synergy activation coefficients H update at every algorithm iteration, according to the following multiplicative update rule:

$$H\leftarrow \;H\;\frac{\left(\;W_{HEALTHY}^{T}\;\cdot \;M\right)}{\left(\;W_{HEALTHY}^{T}\;\;\cdot \;W_{HEALTHY}\;\cdot \;H\right)}$$

where *M* is the matrix of the EMG envelopes measured on the 8 muscles during 20 gait cycles. Each vector of *W*_HEALTHY_ was normalized to unit norm before applying NNR.

Afterwards, the NNR was applied to the EMG envelopes of the specific patient by fixing the synergy activation coefficients H_HEALTHY_ and deriving the patient's weights, using the following update rule:

$$W\leftarrow \;W\;\frac{\left(\;M\;\cdot \;H_{HEALTHY}^{T}\;\right)}{\left(\;W\;\cdot \;H_{HEALTHY}\;\cdot \;\;H_{HEALTHY}^{T}\right)}$$

#### Identification of the patient's impaired muscle synergies

Each of the four reconstructed patient's muscle synergies were compared to the mean healthy synergies by computing the following metrics:

- the similarity between the patient's reconstructed weights and W_HEALTHY_.
- the circular cross correlation computed between the patient's reconstructed activation profiles and H_HEALTHY._
- the time lag computed as: $Tlag=1-|\frac{lag}{100}|\;$, where *lag* is the percentage of gait cycle (lag value can vary between −50 and 50) in which the maximal correlation between the reconstructed activation profile and H_HEALTHY_ was obtained_._
- the activation duration was defined as: $Act=1-\frac{\left|Act_{Hp}-Act_{H_{\text{healthy}}}\right|}{100},$ where Act_Hp_ and *Act*_H_healthy__ are the durations, in percentage of the gait cycle, of the activation phases. These were computed on the patient's reconstructed activation profile and on the mean activation profile of the healthy group, respectively. The activation duration was defined based on the onset and offset values, which were determined from the activation profile using a threshold fixed at the minimum of each profile plus 20% of the cycle peak-to-peak amplitude.

For all metrics a value close to 1 indicates a behavior similar to the healthy subjects. The metrics were first computed on the group of healthy subjects in order to obtain the normality ranges and the specific thresholds to be used to discriminate the impaired muscle synergies. For each metric, a cut-off point of the mean −2·*SD* was chosen to define a threshold common to all muscle synergies. A patient's muscle synergy was defined as impaired when at least one of the metrics resulted under threshold.

#### Definition of the personalized biomimetic stimulation strategy

The individual muscle activations were reconstructed from the representative physiological muscle synergies by multiplying the mean muscle weights and the mean activation profiles of the synergies that resulted to be impaired in the patient as follows: EMG _N × 100_ = W_healthy N × J_^*^ H_healthyJ × 100_ where *N* is number of considered muscles (*N* = 8) and *J* the number of impaired synergies (*J* ≤ 4).

To avoid excessive fatigue due to FES, the stimulation of each muscle was set to zero when the profile was lower than a threshold defined as the value of the baseline plus the 20% of the peak-to-peak amplitude. Finally, when muscles were characterized by very similar activation profiles, if possible, they were grouped to be activated by a single stimulation channel using stimulation electrodes covering both muscles. The stimulation strategy modulated the stimulation pulse width between 0 and a predefined maximum value of 400 μs. The stimulation frequency was common to all muscles and was set to 20 Hz, whereas the stimulation amplitude was identified individually for each muscle, during an initial calibration procedure, in order to produce a visible contraction without discomfort. To identify the stimulation amplitude a pulse width of 400 μs was used.

The control system adapted the predefined biomimetic stimulation strategy to changes in walking speed within session. Indeed, when a subject entered a new gait phase, the average of its duration over the last five strides was computed. This estimate was used to stretch or expand the corresponding part of the stimulation profile in order to fully adapt to the subject's gait timing.

### A preliminary evaluation of the FES treatment effect on two chronic stroke patients

Two patients with chronic hemiparesis due to ischemic stroke (Table 1) were asked to undergo a 4-week intervention consisting of 30-min sessions of FES-supported treadmill walking three times per week. Each session consisted of 5 min of warming up without FES, 20 min of gait supported by the multi-channel personalized FES controller, and 5 min of cooling down without FES. The patient was asked to select his preferred walking velocity during the warming up phase. Before and after the end of the intervention, two clinical scales were assessed: the motor sub-scale of the Functional Independence Measure (FIM) which evaluates the patient's motor disability during daily life activities and ranges from 13 to 91 (independent), (Kidd et al., 1995) and the Mini Best test (MBT) which evaluated the dynamic balance and ranges from 0 to 28 (normal balance; Franchignoni et al., 2010). To evaluate specific improvements in terms of walking ability, the same test used to identify the patient's impaired muscle synergies was repeated at the end of the intervention. Both kinematics and EMG data were collected. The mean cadence was computed from the kinematics data. EMG envelopes were computed and the NMF algorithm was applied to extract the muscle synergies as previously described for healthy subjects. At the end of the intervention, the patients were also asked to rate the global perceived effect of the treatment using the global rating change (GRC), which is an 11-point scale (−5 = made things worse; 0 = not changed; 5 = completely recovered; Kamper et al., 2009).

**Table 1:** **Patient details**.

	**Age (years)**	**Gender**	**Time from stroke**	**Hemiparetic side**
S1	67	Man	11 years	Left
S2	64	Man	9 months	Right

The protocol was approved by the Central Ethics Committee of the Fondazione Salvatore Maugeri (IRCCS) and both patients provided their written informed consent before participation.

## Results

### Functioning of multi-channel synergy-based stimulation controler

#### Healthy muscle synergies

**Figure 3:**
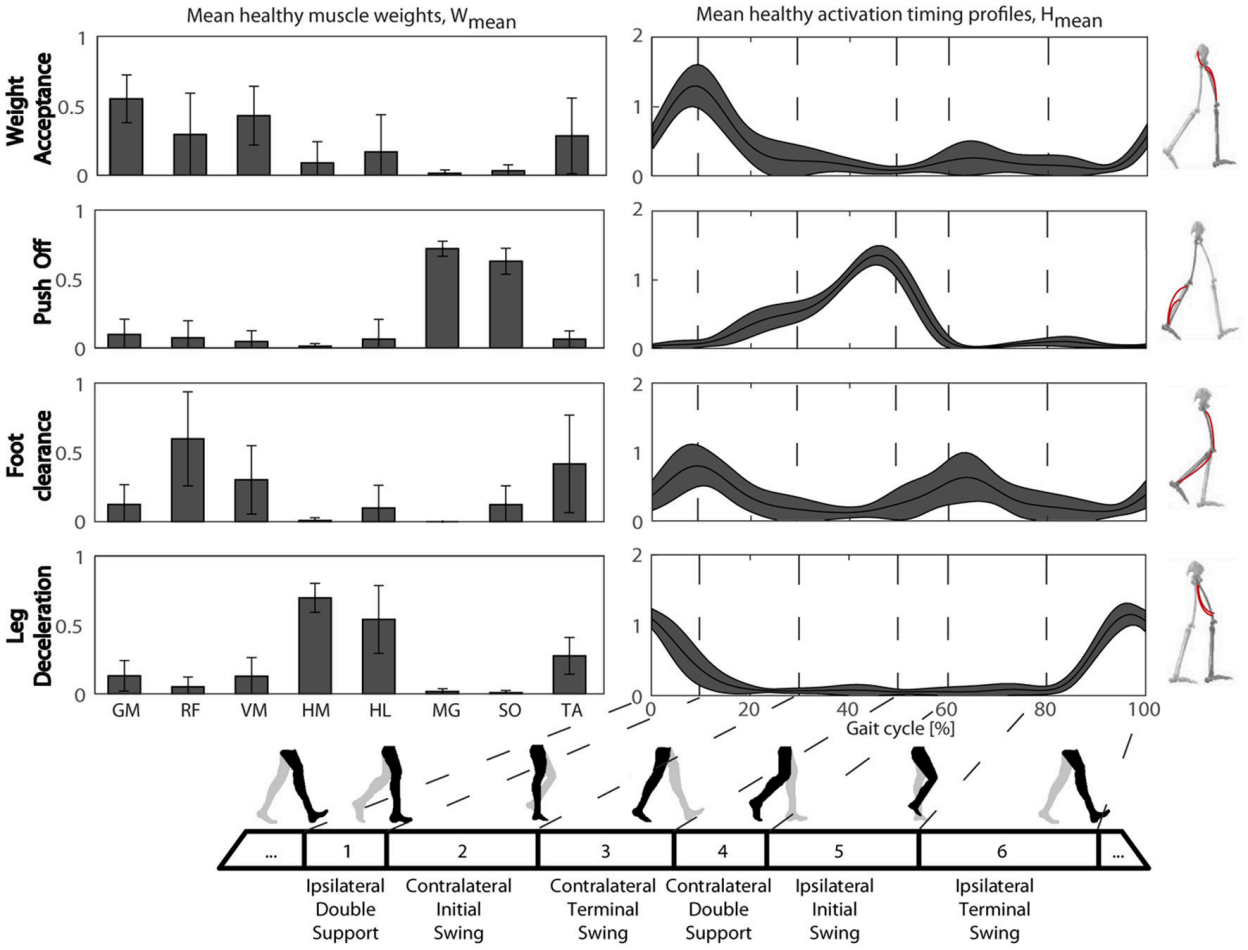
**The physiological muscle synergies: muscle weights (Left panel) and temporal activation profiles (Right panel) obtained during overground walking**. Mean values and standard deviation are reported in both panels. GM, gluteus maximus; RF, rectus femoris; VM, vastus medialis; HM, hamstring medialis; HL, hamstring lateralis; MG, gastrocnemius medialis; TA, tibialis anterior.

The W_HEALTHY_ and H_HEALTHY_ during overground walking are reported in Figure 3. All healthy subjects were characterized by four muscle synergies corresponding to the four gait sub-functions identified in literature: Weight Acceptance (WA), Push Off (PO), Foot Clearance (FC) and Leg Deceleration (LD). The same modular organization, both in terms of spatial composition and temporal recruitment, was obtained during treadmill walking. Indeed, comparing the extracted muscle synergies in the two walking conditions and averaging across subjects, a mean (Standard Deviation, SD) similarity of 0.89 (0.11), a circular cross correlation of 0.94 (0.06), and a time lag of 2 (1) in percentage of the gait cycle were found. This confirms that the two walking conditions share the same muscle coordination and thus it is possible to define both a treadmill and an overground treatment based on the same set of physiological muscle synergies.

The synergies extracted from the overground walking condition were used to perform the two NNR of the EMG envelopes obtained on the paretic side during the patient's pre-treatment assessment.

#### NNR of the patient's EMG envelopes with the healthy synergies

**Figure 4:**
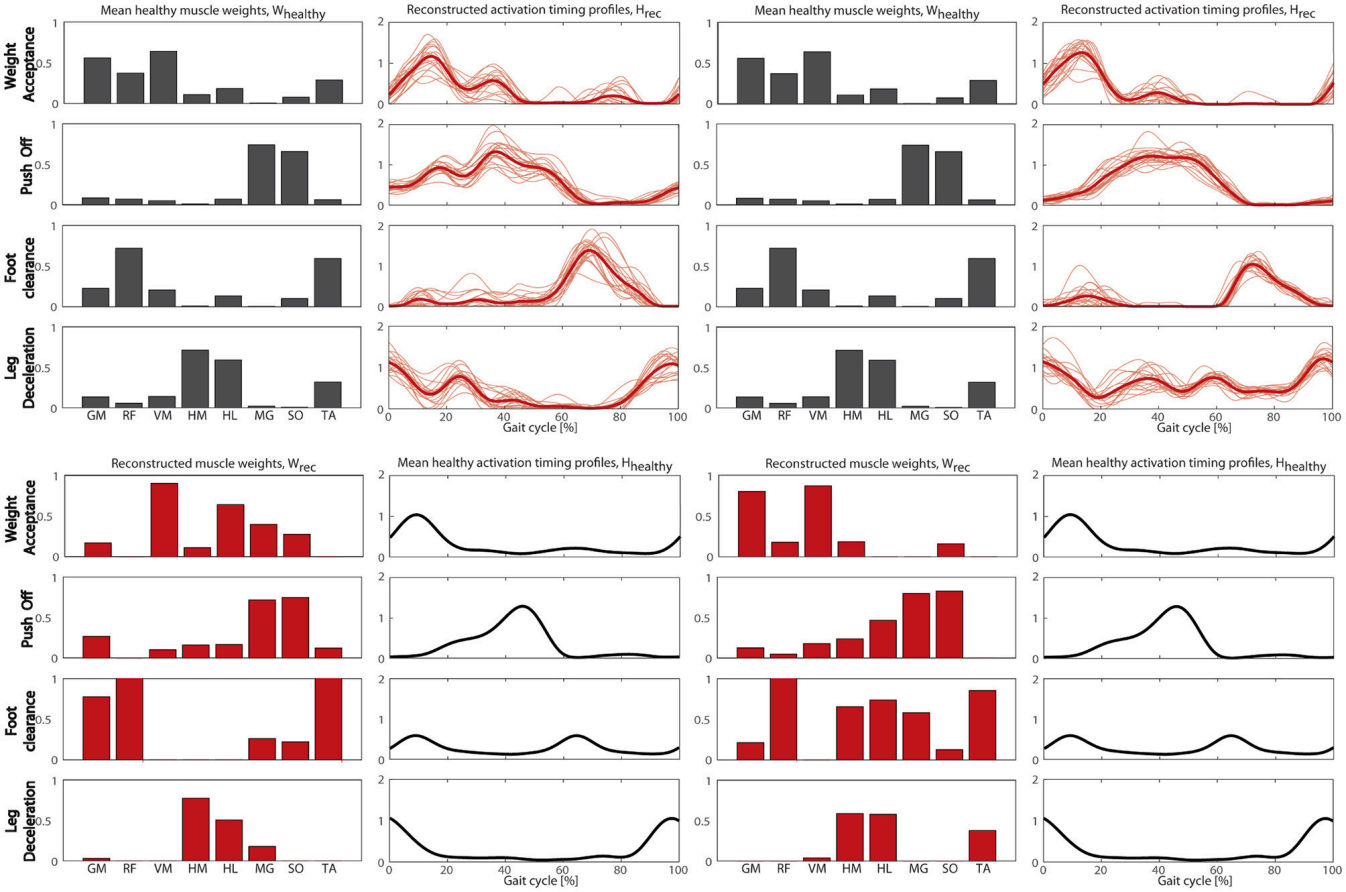
**The reconstructed muscle synergies obtained for S1 (Left panels) and S2 (Right panels) using W_HEALTHY_ (Upper panels) of H_HEALTHY_ (Lower panels)**. In both NNR results, the fixed component is shown in black and the reconstructed one in red. When the activation profiles are reconstructed, both the single-stride profile (thinner lines) and the mean profile (thicker line) are reported.

Figure 4 shows the NNR results obtained for both patients. The obtained VAF values were 0.85 and 0.77 for S1, and 0.90 and 0.84 for S2 when the NNR was applied fixing W_HEALTHY_ and H_HEALTHY_, respectively.

#### Identification of the patient's impaired muscle synergies

Table 2 reports for each metric and each muscle synergy, the thresholds computed on the healthy subjects group (last column) and the values obtained by the two patients during the pre-treatment assessment.

**Table 2:** **Metrics computed on the reconstructed muscle synergies of S1 and S2 for each muscle synergy**.

	**WA**	**PO**	**FC**	**LD**	**Thresholds**
**S1**					
Similarity	**0.67**	0.97	0.95	0.91	0.79
Correlation	0.95	**0.89**	**0.77**	0.92	0.90
T-Lag	0.94	0.96	**0.61**	0.99	0.96
Activation	0.85	**0.71**	**0.65**	**0.77**	0.82
**S2**					
Similarity	0.90	0.93	0.80	0.98	0.79
Correlation	0.96	0.93	**0.79**	**0.85**	0.90
T-Lag	0.96	0.98	0.99	0.99	0.96
Activation	0.99	0.83	0.97	0.99	0.82

The last column indicates the thresholds obtained in the group of healthy subjects. The values below the predefined threshold are highlighted in bold.

The metrics confirmed what was visually observed by the reconstructed synergies: S1 had an impaired spatial composition in the WA synergy (similarity was under threshold for WA), a wider activation timing of PO and LD synergies, and a delayed recruitment of the FC synergy. Concerning S2, a low cross correlation was found for FC and LD synergies. Thus, all four synergies were defined as impaired for S1 and only FC and LD were considered impaired synergies for S2.

#### Definition of the personalized stimulation strategy

**Figure 5:**
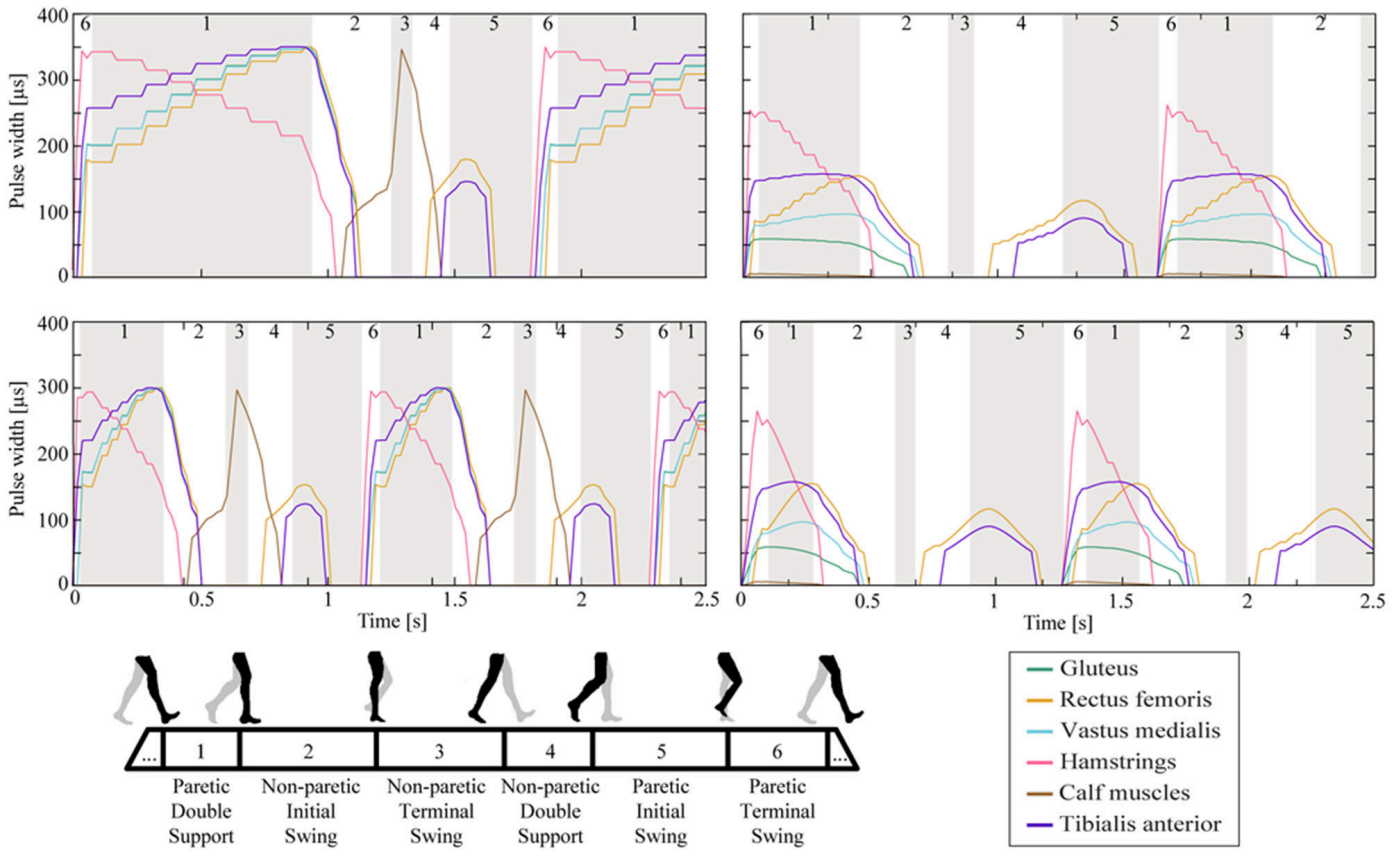
**The personalized stimulation strategy obtained for S1 (Left panels) and S2 (Right panels) in the first (Upper panels) and last (Lower panels) sessions of the intervention**. Same labels as in Figure 3.

The final FES strategy obtained and used for both patients is shown in Figure 5. The medial and lateral hamstrings and the medial gastrocnemius and soleus showed similar activation profiles and therefore the FES strategy coupled into one stimulation channel both hamstrings, and the calf muscles into another. Thus, a total of six muscle groups were stimulated.

S1 had a FES strategy based on all four healthy synergies, whereas for S2 only the FC and LD synergies was used to obtain the muscle stimulation profiles. When a reduced number of synergies was used to create the stimulation strategy, a subset of the six muscle groups was stimulated. In particular, the calf muscles, which were not recruited by the FC and LD healthy synergies, were not stimulated for S2.

Figure 5 also shows the different kinematic patterns of the two patients. Indeed, both patients were characterized by a prolonged double support, but S1 extended the paretic double support phase (gait phase 1) whereas S2 extended the non-paretic double support phase (gait phase 4). In both cases, the FES strategy was able to adapt to these changes in gait pattern, mapping the stimulation profiles accordingly.

For both patients the first (upper panels) and last (lower panels) sessions of the intervention are shown to highlight the differences in gait speed together with slight differences in the kinematic pattern. In the first session of FES-supported gait, S1 presented an impaired kinematic pattern characterized by a double support phase of the paretic leg (phase 1) lasting the 48% of the gait cycle and a very short paretic single support (21% of the gait cycle). In the last treatment session, this kinematic pattern changed: the duration of the paretic double support was the 25% of the gait cycle and the duration of the paretic single support was 29%. These improvements in the kinematic pattern corresponded to a walking speed that in the last session doubled its value with respect to the first session. Concerning S2, the kinematics pattern in the first session was characterized by a reduced paretic swing phase that was augmented by 52% in the last session, achieving a final duration equal to the 38% of the gait cycle.

### The FES treatment effect on the two chronic stroke patients

Both patients completed the treatment without difficulties and reported a positive global perceived effect of the treatment (GRC was +4 (improved a lot) and +2 (improved) for S1 and S2, respectively). The treadmill speed used in the first and last day of treatment increased from 0.43 to 0.83 m/s, and from 0.38 to 0.68 m/s for S1 and S2, respectively.

**Figure 6:**
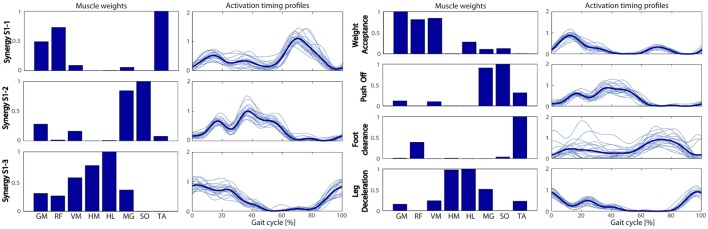
**The extracted muscle synergies obtained for S1 before (Left panel) and after (Right panel) treatment**. Same labels as in Figure 3.

**Figure 7:**
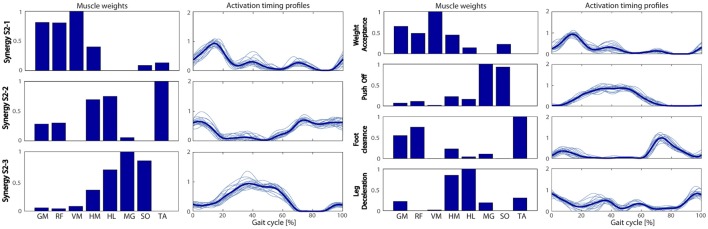
**The extracted muscle synergies obtained for S2 before (Left panel) and after (Right panel) treatment**. Same labels as in Figure 3.

The extracted muscle synergies before and after the treatment during the overground walking tests are shown in Figures 6, 7 for S1 and S2, respectively. The treatment induced an increase of the number of extracted synergies in both patients from 3 to 4 indicating a general improvement in muscle coordination. The VAF was 0.87 before and 0.88 after treatment, and 0.90 before and 0.93 after treatment for S1 and S2, respectively. The visual comparison between the extracted synergies obtained before intervention for S1 and the healthy synergies (Figure 6) suggests that the first extracted synergy (S1-1) resembles the FC synergy except for the GM activation, the second extracted synergy (S1-2) mostly recruits the MG and SO muscles as it is in the PO synergy with an anticipatory activation profile, and the third synergy (S1-3) merges the WA and LD synergies. After treatment, four muscle synergies were found, generally resembling the spatial composition of the healthy muscle synergies in Figure 3. An early recruitment of the plantar-flexors is still present in the PO synergy.

Concerning S2 (Figure 7), before treatment the first synergy (S2-1) can be associated to the WA synergy, the second (S2-2) seems to be the merging of the FC and LD synergies, and the third (S2-3) seems to be the PO synergy with a slight contribution of the hamstring muscles. After treatment, four synergies were obtained displaying a behavior more similar to healthy subjects; however, an early activation of the plantar-flexors is still visible in PO synergy.

The clinical evaluation of the two patients is reported in Table 3. Both patients had a mild motor disability at baseline. In both cases the improvement gained in muscle coordination was not yet enough to be transferred into a significant difference in general motor disability.

**Table 3:** **Comparison of the outcome measures before and after training**.

	**S1**		**S2**	
	**T0**	**T1**	**T0**	**T1**
Mini Best Test (0–28)	17	22	21	23
FIM motor subscale (13–91)	78	78	85	85
Cadence (strides/s) (SD)	0.98 (0.03)	1.01 (0.03)	0.81 (0.03)	0.80 (0.02)

## Discussion

The high heterogeneity of stroke patients and the high variability in their response to treatments demand for novel personalized assessment methodologies able to unveil the specific impaired sub-functions to be recovered and for novel training procedures adapted to single subject's disability. Most of the outcome measures used to assess the patients' condition are focused on the overall motor function (such as the walking speed) and do not have the power to discriminate specific impairments that underlie the general functional deficit (Ting et al., 2015). Recent neurophysiological studies recommended the analysis of muscle synergies to finely assess the impairment of the subject, to design rehabilitation treatments personalized on the specific nature of the individual impairment, and to assess the eventual treatment outcome (Clark et al., 2010; Ting et al., 2015). In this scope, we developed a multi-channel FES controller to support gait training based on physiological muscle synergies and we personalized it to the individual impairment obtained by means of a baseline assessment of gait muscle synergies. The proposed treatment is goal-oriented, task-specific, and challenging, since the subjects are asked to walk for 30 min at a comfortable speed. FES patterns have been accurately aligned in time with the gait phases of the subject so to assure the maximal synchronization between the FES input and the volitional activity. Thus, it should harness activity-dependent neuro-plasticity (Lamontagne and Fung, 2004; Gandolla et al., 2014).

In literature, FES controllers have been based on very simple segmentation algorithms able to discriminate between the stance and swing phases and the FES strategy has been linearly mapped through the gait stride adopting sub-optimal time rules in order to automatically deactivate stimulation (Daly et al., 2011). The novelty of our proposed control system lies in the capability to accurately map the subject's gait timing based on the real-time detection of six gait phases. This mapping is able to stretch or extend the stimulation profiles according to the actual duration of all six phases. For instance, if a patient's gait pattern is characterized by an extended double support phase, the stimulation profile of the muscles supporting this phase are extended accordingly in order to follow the correct muscle timing and coordination involved in this phase (see Figure 5). This choice avoids the unwanted activation of antagonist muscles in the extended kinematic phase that could increase the instability of the gait instead of fostering the relearning process. This novel modality to map the individual kinematics is an important personalization factor, since the duration of each gait phase is highly dependent on the patient's level of impairment (Olney and Richards, 1996).

The starting point to design the proposed biomimetic multi-channel FES controller was the identification of the physiological set of synergies during walking. Results obtained in healthy subjects confirmed that a common motor-control strategy based on four muscles synergies, was shared across walking conditions (overground and treadmill). The identified muscle synergies corresponded well to the four biomechanical functions proposed in the literature (Clark et al., 2010) and confirmed the equivalence in motor coordination between the two walking conditions (Kautz et al., 2011). The physiological muscle synergies used to design the stimulation strategy were extracted from a group of healthy subjects, which were younger than the average stroke patient; nevertheless, it has been shown that synergies are invariant with aging (Monaco et al., 2010).

The two patients included in the pilot study were quite independent in daily-life activities before treatment and were characterized by a mild gait disability (gait speed higher than 0.8 m/s; Tilson et al., 2010). However, a more specific analysis based on muscle synergies during overground gait highlighted an altered motor coordination characterized by only 3 independently recruited muscle synergies for both patients. The underlying gait abnormality was different for the two patients. S1 merged the control of the proximal extensors with the hamstrings (i.e., the WA and LD synergies) and showed an early activation of the plantar-flexors, which is a typical behavior of stroke patients (Clark et al., 2010). S2 merged the FC and LD synergies and also showed an early recruitment of the plantar-flexors presumably connected to an increased excitability of the monosynaptic stretch reflex (Crenna and Frigo, 1987). These two different impairments in terms of muscle coordination produced a proper personalized treatment that resulted in a FES strategy based on all 4 or just 2 muscle synergies for S1 and S2, respectively. Comparing the first and last session of treatment (Figure 5), a different kinematic pattern is noticeable. The first patient was able to reduce by half the duration of his paretic double support phase. This represents a big improvement although it was still higher than the physiological duration, which is about 10% of the gait cycle (Perry and Burnfield, 1992). Analogously, the paretic single support increased by 38% becoming closer to the physiological value (Perry and Burnfield, 1992). The second subject improved the duration of his paretic swing phase. For both subjects the improvements across treatment sessions in gait timing were coupled with a faster speed in the training execution.

The assessment after the end of the intervention showed that both patients improved their muscle coordination; indeed, four muscle synergies were extracted for both of them. The originally merged synergies seemed to regain their distinct role in locomotion control, even if the PO synergy maintained an anticipated recruitment and a prolonged timing in both patients. The benefits of the treatment were more evident for S1 and this was confirmed by the patient's perceived effect; indeed, S1 stated that, thanks to the treatment, he was able to achieve a very good walking improvement (GRC = 4). The improvement in motor coordination was also coupled with a clinically important improvement in the dynamic balance. Indeed, the pre-post change of the Mini Best Test overcame 4 points (Table 3) that is the minimal clinical important difference for patients with balance disorders (Godi et al., 2013). Concerning S2, the treatment was able to induce a beginning of neuro-motor relearning, but probably the 12 treatment sessions were not enough to translate the motor-coordination progress into relevant clinical improvements, in line with the lower benefit perceived by the patient (GRC = 2).

The duration of the donning and setting up of the FES controller was performed by the physiotherapists and, excluding the first day of training, it lasted an average of 5–10 min, which was compatible with a clinical use.

This study has two main limitations. First, the treatment was tested only with two chronic stroke patients and the results obtained, even if encouraging, should be considered as preliminary. Second, the speed used during training was too slow. For instance, the second subject never reached his self-selected speed overground during training. Recent studies demonstrated that combining a 2-channel FES with fast treadmill walking yielded larger improvements in gait mechanics than when FES was combined with self-selected speed treadmill walking (Kesar et al., 2011; Awad et al., 2016). Thus, future studies using this personalized biomimetic FES controller should consider the use of fast speed during training.

## Conclusion

In this study we have developed and tested a personalized multi-channel FES controller to support gait rehabilitation after stroke. The treatment was personalized to the specific gait abnormality of each patient. Indeed, once the impaired biomechanical functions were revealed by an assessment based on muscular synergies analysis, the exercise was shaped in order to train only the muscle coordination associated with those biomechanical functions. The muscle-synergy analysis was also exploited to assess the effects of treatment and confirmed to be very effective in identifying improvements in motor coordination. The results presented in this pilot case study were encouraging; however, they should be confirmed by a wider statistical study (e.g., a randomized controlled study). Additionally, the application of this multi-channel FES controller could be extended to people with post-acute stroke, whose lack of a well-learnt compensatory strategy by the CNS could help improving the benefits obtained with the proposed treatment in case of chronic patients.

## Author contributions

SF designed the work, drafted the manuscript, refined the data analysis, interpreted the results, revised and approved the manuscript. NC designed the work, collected the data, carried out the data analysis, interpreted the results and revised and approved the manuscript. EA designed the work, interpreted the results, revised and approved the manuscript. AN designed the work, recruited the patients, supervised the intervention, revised and approved the manuscript. AT performed the clinical scale assessment, carried out the intervention, revised and approved the manuscript. MM revised and approved the manuscript. GF designed the work, revised and approved the manuscript. AP designed the work, revised and approved the manuscript.

## Funding

The work was partially supported by the PRIN project titled “Fall risk estimation and prevention in the elderly using a quantitative multifactorial approach” (grant no.: 2010R277FT) and partly by the project titled “Fall prevention and locomotion recovery in post-stroke patients: a multimodal training towards a more autonomous daily life” funded by the Italian Ministry of Health (grant no.: GR-2010-2312228).

### Conflict of interest statement

The authors declare that the research was conducted in the absence of any commercial or financial relationships that could be construed as a potential conflict of interest.
